# Trends in physical and mental health needs across generations in Australia

**DOI:** 10.1007/s10198-025-01791-6

**Published:** 2025-07-03

**Authors:** Sabrina Lenzen, Luke Connelly, William Whittaker, Stephen Birch

**Affiliations:** 1 Centre for the Business and Economics of Health, The University of Queensland, QLD, St Lucia, 4072 Australia; 2 Department of Sociology and Business Law, The University of Bologna, 40126 Bologna, Italy; 3 Institute of Population Health, Public Health, Policy and Systems, University of Liverpool, Liverpool, UK; 4 Division of Population Health, Health Services Research & Primary Care, The University of Manchester, Manchester, UK; 5 Department of Clinical Epidemiology and Biostatistics, McMaster University, Ontario, Canada

**Keywords:** Health service planning, Needs-based, Mental health, Birth cohorts, HILDA, I10, I18, J11

## Abstract

Despite evidence of changes in age-specific incidence and prevalence rates for chronic conditions and disease, future health resource planning is often based on historical age- and gender-specific service use, neglecting changes in the need for care within age groups between generations. This paper studies differences in health needs by age and gender across birth cohorts in Australia and considers the implications for future health service planning. Whilst controlling for age and period effects, we find that more recent-born female birth cohorts have higher prevalence rates of long-term health conditions than earlier-born cohorts, whereas we don’t find an effect for males. The increase for females corresponds with an increase in probable mental disorders, and while we also find an increase in probable mental disorders among males, decreases in physical impairment rates among both genders offset the overall rates of long-term health conditions among males but not among females, where increases in probable mental disorders are larger. Comparing projections of mental health service requirements that integrate cohort effects, as opposed to those that do not, shows that traditional planning models may underestimate health service requirements for the future. Our findings suggest that health service planners should relax assumptions about constant age-specific use.

## Introduction

Health service planning is pivotal in making the most effective use of available and future health care resources and in order to plan appropriately for recruitment and training of health care professionals to meet future population needs. An aspect that is often neglected in health service planning models is the consideration of changes in age-specific prevalence and incidence rates of disease and disability over time, i.e., changes in health care needs by age. For example, 65-year-olds in 2023 have very different health profiles than 65-year-olds in 1950. But despite this evidence, most governments base their health service plans on historical age-specific health service use estimates and apply this number to future population estimates in the respective age groups [26, 27]. In other words, it is assumed that previous age-specific use rates will continue.

Planning based on historical use is problematic for two reasons. First, the population’s current use of health services does not necessarily meet that population’s needs. In fact, there is much evidence of unmet health care needs for certain conditions, or subgroups of people, such as those on lower incomes or from disadvantaged backgrounds as well as for people living in more rural as opposed to urban areas [15, 22]. At the same time, it has been shown that in other areas health services are being overutilised [6]. A mismatch of demand and supply in health care has been recognised by economists for several decades [17]. Using historical use estimates for planning for the future will therefore perpetuate current problems into the future, instead of improving unmet care needs.

Second, even if current health service use were to meet the needs of populations perfectly, the need for health care is changing, over time, both within and between age groups. For example, while overall dementia prevalence rates are predicted to increase over the next decades, due to an increase in population numbers in old age, age-specific dementia incidence rates are declining in more recent birth cohorts [13, 18, 24]. At the same time, some evidence suggests that more recent birth cohorts will have a shorter life expectancy than the current generations due to the impacts of obesity and sedentary lifestyles, changing health care needs at different ages [2]. We argue that incorporating these changing health care needs into health service planning is crucial to better align health care supply with needs in the future.

In the existing literature, advantages of needs-based planning models have been discussed in England and Canada, where the authors highlight the importance of accounting for birth cohort effects when estimating future health care needs [21, 30]. Lenzen and Birch [19] discuss the advantages of dynamic needs-based health service planning models in the Australian context, but do not account for period effects in their analysis. In addition, when attempting to incorporate changes in need over time and between cohorts, gender specific differences in need should be taken into consideration. Research has shown that prevalence estimates of disease and disability do differ substantially by gender. For example, while females are more likely to develop conditions such as dementia in old age [20], the prevalence of coronary heart disease is much higher among males than females [31]. Neglecting any of these gender-specific differences in health needs and taking into consideration the greater longevity of females substantially affects future health service use estimates.

We add to the literature in several important ways. Foremost, this is the first study to investigate changes in health needs across birth cohorts, stratifying need estimates not only by age but also by gender, and the first study to do so for the Australian population. Using data over 21 years from a representative sample of the Australian population, we are able to address the following research questions: How do health needs differ across birth cohorts after controlling for age and period? How does incorporating gender-specific cohort effects into future health need predictions compare to traditional age-constant models? In addition, we are the first to provide needs-based predictions for future mental health service requirements by estimating future prevalence rates of mental disorders, applying dynamic age- and cohort-specific predictions, and multiplying these with service requirements.

## Methods

### Empirical methodology

#### Health service planning models

In conventional health service planning models, future health service requirements are based on historical age-specific use estimates and projected onto the future population size. In other words, future health service requirements ($$HealthCar{e}_{t+1})$$ are a function of the future projected population size $$(Populatio{n}_{a,t+1})$$ in each age group (*a*) and the current or past mean level of health care use per capita $$(HealthCar{e}_{a,t})$$ in that age group:

1 $$HealthCar{e}_{t+1}=\sum\limits_{a=1}^{N}Populatio{n}_{a,t+1}*HealthCar{e}_{a,t}$$

where a represent age groups (a = 1, 2, 3, …, n). *t* indicates the current year, and *t* + *1* any future years. In practice, levels of health care use by age group can be estimated using individual level survey data and linear regression models of health care use as the dependent variable, regressed on age dummies and a random error term (${\upepsilon }_{\mathrm{i},\mathrm{t}}$) as follows:

2 $$HealthCar{e}_{i,t}={\updelta }_{0}+{\upbeta }_{1}agegrou{p}_{a,i,t}+{\upepsilon }_{i,t}$$

where i indexes individuals and t indexes time.

While some planning models include more sophisticated versions of this model, such as focusing on previous trends in use, their estimates remain based on utilisation rates rather than need, thus perpetuating a continuation of the potential mismatch between use and needs [27].

As a solution to this problem, we suggest an approach in which future health service requirements are based on predicted future age- and cohort-specific health needs, as follows:

3 $$HealthCar{e}_{t+1}=\left(\sum\limits_{c=1}^{{N}{\prime}}\sum\limits_{a=1}^{N}Populatio{n}_{a,t+1}*\widehat{HealthNee{d}_{a,c,t+1}}\right)*\frac{HealthService{s}_{t+1}}{HealthNee{d}_{t+1}}$$

here c represents corresponding birth cohorts $(c=1,2,3...n')$ and ${Health\widehat{Need}}_{a,c,t+1}$ are predicted age *(a)* and birth cohort *(c)* specific estimates of health needs based on prevalence rates of health conditions or in our example health need proxies. $\frac{{HealthServices}_{t+1}}{{HealthNeed}_{t+1}}$ represent the predicted amount and type of services that are required to meet the population’s predicted health needs. The aggregated amount of health service requirements is thus a function of health needs estimates per age group, multiplied by population predictions of the respective age group and service requirements to meet that population’s health needs.

#### Predicting health needs

While Eq. (3) is straightforward, understanding changes in health needs across age and cohort is complex and tackling these methodological issues is the focus of our analysis. We start by estimating age-specific health needs, similar to what is done in traditional health service planning models, but instead of using health care as dependent variable we use health needs proxies:

4 $$HealthNee{d}_{i,t}={\updelta }_{1}+{\upbeta }_{2}agegrou{p}_{a,i,t}+{\upepsilon }_{i,t}$$

Changes in the need for health care by age groups over time though are not incorporated in Eq. (4) and therefore as a next step, we include a time trend or year dummies ${d}_{i,t}$ as follows:

5 $$HealthNee{d}_{i,t}={\updelta }_{2}+{\upbeta }_{3}agegrou{p}_{a,i,t}+{\upgamma }_{1}{d}_{i,t}+{\upepsilon }_{i,t}$$

While this model represents an improvement over model (4), it still does not account for birth cohort-specific trends. However, as pointed out in the Introduction, health care needs might not only vary by age and over time but also between birth cohorts. In particular, birth cohort effects might occur due to environmental factors that are related to health but are unequally distributed across a population and across time. For example, younger people today may have higher access to processed foods than younger people of previous generations. Birth cohort effects can also occur because the impact of environmental factors on health may differ across age groups. For example, exposure to high levels of air pollution may affect younger people more than older people. At the same time, health changes over the life course, as people age (age effects); or due to events that happen in a particular year, such as the recent Covid-19 pandemic or medical advances (year effects). Including all three of these variables, however, is problematic, because of the age, period, and cohort identification problem. Even with panel data and a survey period that spans many years, cohort effects are difficult to identify because they are correlated with the age and period effects. In other words, it is not possible to control for age, birth-year, and period/year in the same equation, because age is a perfect linear combination of birth-year and survey period. The challenge we face is how to control for all three dimensions at the same time. We propose to overcome this challenge using two leading approaches in the literature.

First, to resolve any issues of collinearity between age, period and cohort, Deaton and Paxson [11] and Deaton [10] proposed an approach in which the year dummies are transformed to estimate unbiased coefficients on age and cohort. Since we are primarily interested in the cohort effects and not so much in any year or period effects when estimating future healthcare requirements, we find the approach suitable for our research questions. More specifically, Deaton and Paxson [11], proposed that the year dummies be transformed to follow a trend around zero, which still allows to account for any fluctuations over the survey periods, without potentially biasing the age and/or cohort estimates. We follow this approach and transform year dummies in the following way:

6 $${d}_{i,t}^{*}={d}_{i,t}-\left[\left(surveynumber-1\right){d}_{i,surveywave2}-\left(surveynumber-2\right){d}_{i,surveywave1}\right]$$

where ${d}_{i,t}^{*}$ is the transformed year variable, ${d}_{i,t}$ is a binary year variable for individual i at survey year t. surveynumber is the survey number. By construction, ${d}_{i,surveywave1}^{*}$ and ${d}_{i,surveywave2}^{*}$ will equal zero and are thus omitted from the regression. If we include this transformed year variable in the estimated equation for health needs, prevalence rates can be estimated as a function of age, cohort effects, and year effects, reducing potential bias caused by specific time/period effects that may influence some cohorts and ages more than others:

7 $$HealthNee{d}_{i,t}={\updelta }_{3}+{\upbeta }_{4}agegrou{p}_{a,i,t}+{\theta }_{1}cohor{t}_{c,i,t}+{\gamma }_{2}{d*}_{i,t}+{\upepsilon }_{i,t}$$

Second, we will employ a further methodology that has been used in birth cohort analysis. The proxy variables approach uses one or more proxy variables to substitute either the age, period or cohort variable. This is a popular approach because of its intuitive rationale. After all, the age, period, and cohort variables serve as proxies for different sets of unmeasured correlates. In the literature, several papers have used among others, the unemployment rates [14, 28] and GDP growth rate [12, 14, 25, 28] as proxy variables for period effects. In this context, it is important that proxy variables are not linearly related to cohorts or periods since, otherwise, they will be highly collinear with the other two effects, just as the effects they are intended to replace in the models [16].

We propose to follow the literature and use GDP growth rates as a proxy for period effects in robustness checks. Globally, a clear strong positive correlation between health and GDP exists. Countries with better health status tend to have higher incomes than countries with worse health status, a relationship known as the “Preston curve” (see [23]). Similarly, within countries, people with higher incomes tend to have better health than those with lower incomes. This relationship has also been observed over time. While the average life expectancy in 1960 was just over 70 years in Australia, it is now well over 80 years. At the same time, GDP has grown substantially over the same time period [27]. Clearly, this relationship alone does not allow us to conclude that higher income or better economic growth leads to better health outcomes, since the main forces that drive economic growth, such as technological progress, education, and physical capital accumulation also promote population health, but we do not need to be able to disentangle the drivers of this relationship, as long as we believe that they are correlated with GDP.

#### Estimating future health needs

Using previously estimated age and cohort effects, we now estimate future health needs by age group. More specifically, we compare health care needs estimates using (i) only the age effects plus the constant, i.e., ${\updelta }_{1}+{\upbeta }_{2}$, following Eq. 4, with (ii) both the age as well as cohort effects plus the constant, i.e., ${\updelta }_{3}+{\upbeta }_{4}+{\theta }_{1}$, following Eq. 7. The latter accounts for changes in health needs across birth cohorts and is our preferred specification. To estimate the aggregate number of people who will require health care in the future, we multiply the age specific health need prevalence rates with official Australian population projections from the Australian Bureau of Statistics in the respective age group [1]. These estimates can be found in the Appendix, Tables A2 and A3.

#### Estimating future health service requirements: a case of probable mental disorders

As a next step, we use estimated future health needs to predict future health service requirements. Since health service requirements vary depending on the disease or condition, ideally one would want to calculate future health care requirements for every condition separately. While this is out of the scope of a single research paper, instead, we present a case study for probable mental disorder, as a proof of concept. We focus on mental health disorders because among the three health need proxies, we find that probable mental disorders change the most and are therefore most relevant to future health service planning. In addition, HILDA respondents are specifically asked about mental health service use, allowing us to estimate historical use.^1^

First, we follow traditional planning methods and use these historical age-specific use estimates from HILDA and apply them to predicted probable mental disorder prevalence rates for each age group from the previous section. Next, we compare these traditional projections with projections that apply recommended service use based on clinical guidelines. Here, we follow the recent review of evidence-based psychological interventions in the treatment of mental disorders, published by the Australian Psychological Society [4].^2^

### Data

Our analysis uses data from the HILDA Survey which has been conducted annually since 2001. The survey sample is an area-based clustered sample of households initially interviewing almost 14,000 persons from 7,682 households. In 2011, another 4,000 persons from 2,153 households were added to the sample [29]. HILDA is divided into a face-to-face interview as well as a self-completion supplement, including questions about family life, income, and employment, as well as health, and health behaviours.

With the goal to study populations’ ‘overall’ health service needs, we start our analysis with a health need proxy measure that captures whether respondents experience any ‘*long-term health condition, impairment or disability that restricts them in their everyday activities, and has lasted or is likely to last, for 6 months or more’* (see Appendix, Table A1). We assume that this measure is most predictive of the overall need for health care, due to its ‘objective’ nature, and because it is most similar to proxies used in previous studies, such as limiting long-standing illness [21, 30]. When answering this question in the survey, respondents were provided with a list of conditions, including among others sight, hearing and speech problems, difficulty learning or understanding things, limited use of arms, or legs and conditions that impact physical activity or physical work, pain, shortness of breath and any mental illness which requires help or a nervous or emotional condition which requires treatment.

The choice of an appropriate measure of overall health needs is a crucial cornerstone of this paper and one may argue that some health needs are not picked up by measuring only long-term health conditions. We therefore also use two quality-of-life measures as health need proxies. Here we use information from the SF-36 questionnaire that is part of the self-completion part of the HILDA survey. The SF-36 instrument is an internationally recognised diagnostic tool for assessing functional health status and well-being. More specifically, the SF-36 consists of eight scaled scores, which are the weighted sums of the questions in their section. Dimensions include vitality, physical functioning, bodily pain, general health perceptions, physical role functioning, emotional role functioning, social role functioning and mental health. Each scale is directly transformed into a 0–100 scale on the assumption that each question in the respective dimension carries equal weight. We focus on the SF-36 subscales measuring (1) physical functioning, and (2) mental health problems, thus picking up one physical health as well as one mental health dimension. We use dichotomous measures of the variables. Physical impairment is derived from the physical functioning subscale, identifying someone as being in poor physical health if the respondent scores below 68 on the transformed scale. This equals one standard deviation below the mean and has been used in previous studies [8]. Probable mental disorder has been defined as respondents scoring below 52 on the mental health scale in several previous studies, which we use in our analysis by creating a dichotomous measure of mental health [7, 9].

Having 21 years of data, we divide our sample into 12 seven-year age groups (15–21 up to 92 and above) and 12 seven-year birth cohorts (1923–1929, … 2000–2006). With seven being a divisor of 21, we are able to split our sample into these equally sized age-cohort combinations. The sample sizes for people born before 1923 are very small and are therefore not part of our analyses. Tables A4 and A5 provide sample sizes for all other birth cohort age group combinations over the study period for our female and male sample, respectively.

We estimate the rate of health needs, i.e. prevalence rates of health need proxies, in each age group for the years 2000, 2007, 2014, 2021, 2028, and 2035. The years are chosen based on corresponding birth cohort-age-group combinations in our dataset. For example, 22- to 28-year-olds in 2000 were born between 1972 to 1978. An overview of these matching year, age group and cohort combinations can be found in Table A6. Since we are missing cohort estimates for people aged 70 and above in 2000, 77 and above in 2007 and 85 and above in 2014, our results will focus on comparing 2021, 2028, and 2035 estimates. This is in line with our research question, considering that we are interested in health service projections for the future. For the youngest age.

group in 2035, we do not have a cohort effect estimate either, so we replace it with the cohort effect of 2000 − 2006.

## Results

### Descriptive statistics

Our full sample contains 318,608 observations with 151,133 male and 167,475 female observations. Tables A7 and A8 contain the percentage of females and males in each age-group-cohort combination that have at least one long-term health condition. Statistics for physical impairment and mental disorder prevalence rates by age and cohort are provided in Tables A9 to A12 in the Appendix. As expected, particularly younger age groups have low shares of their population experiencing long-term health conditions, and that increases starting from age 50–60. For example, the average prevalence of respondents with long-term health conditions is around 24 percent among 43 to 49-year-old females, but this increases to 40 percent among 57 to 63-year-old females. The reverse is true for mental disorders, though, where prevalence rates are higher among younger age groups. Among young females, the prevalence of probable mental disorders is 17 percent for 15 to 21-year-olds, compared to 11 percent among 57 to 63-year-old females. We observe lower rates among males, where the prevalence is 10 percent among 15 to 21-year-olds and eight percent among 57 to 63-year-olds.

### Cohort Effects

**Fig. 1:**
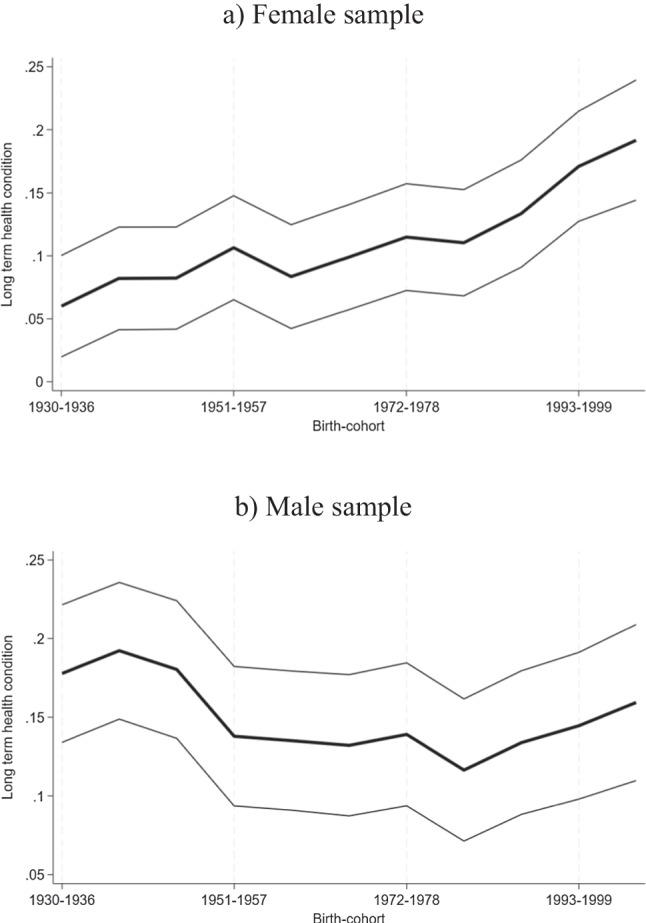
Plotted birth cohort effects of long-term health conditions for females and males after controlling for age and period

We now present the results of our main analyses. Figure 1 plots the estimated cohort effects based on Eq. (7), using long-term health conditions as a proxy for health care needs for females and males, respectively. The corresponding regression results can be found in Table 1 for the models (i) age only, based on Eq. 4 and (ii) age, cohort, and period, based on Eq. 7. Columns (1) and (2) present results for females, whereas columns (3) and (4) present results for males. The plotted cohort effects correspond to columns (2) and (4) in Table 1. For all models, the age group 15–22 and the birth cohort 1922–1929 are the reference categories. The prevalence of long-term health conditions increases among females born more recently, after controlling for age and period. Except for the cohort closest to the reference, all other cohort coefficients are statistically significant. For males, the signs are negative but statistically insignificant, except for the 1979 to 1985 cohort, which shows a statistically significant reduction of long-term health conditions by about five percent. Our results are robust, when using the proxy variable method, rather than the Deaton and Paxson [11] method. However, the reduction in long-term health conditions for males among more recently born birth cohorts is statistically significant for most cohorts using the proxy variable approach. Results can be found in the Appendix, Table A14.

**Table 1:** Age and/or cohort effects on long-term health conditions among male and female respondents in HILDA between 2001 and 2021

	Female sample		Male sample	
	(1)	(2)	(3)	(4)
	Age only	Age, Cohort, Period	Age only	Age, Cohort, Period
Age group				
*Reference group: Respondents below 22 years of age*				
22—28	0.019*** (0.004)	0.029*** (0.005)	0.014*** (0.004)	0.016*** (0.004)
29—35	0.040*** (0.005)	0.061*** (0.006)	0.039*** (0.005)	0.041*** (0.006)
36—42	0.060*** (0.006)	0.088*** (0.007)	0.066*** (0.006)	0.065*** (0.007)
43—49	0.114*** (0.006)	0.147*** (0.009)	0.114*** (0.006)	0.111*** (0.009)
50—56	0.187*** (0.007)	0.224*** (0.010)	0.172*** (0.007)	0.164*** (0.010)
57—63	0.266*** (0.007)	0.308*** (0.011)	0.251*** (0.008)	0.239*** (0.011)
64—70	0.335*** (0.008)	0.383*** (0.012)	0.330*** (0.008)	0.313*** (0.013)
71—77	0.447*** (0.009)	0.501*** (0.014)	0.414*** (0.010)	0.393*** (0.015)
78—84	0.582*** (0.011)	0.639*** (0.017)	0.557*** (0.012)	0.535*** (0.018)
85—91	0.728*** (0.017)	0.791*** (0.022)	0.682*** (0.020)	0.660*** (0.024)
92—older	0.836*** (0.037)	0.907*** (0.040)	0.772*** (0.063)	0.751*** (0.065)
Birth year				
*Reference group: Respondents born before 1930*				
1930—1936		0.031 (0.021)		0.016 (0.022)
1937—1943		0.053** (0.021)		0.030 (0.022)
1944—1950		0.054*** (0.021)		0.018 (0.022)
1951—1957		0.078*** (0.021)		−0.024 (0.023)
1958—1964		0.055*** (0.021)		−0.027 (0.023)
1965—1971		0.070*** (0.021)		−0.030 (0.023)
1972—1978		0.086*** (0.022)		−0.023 (0.023)
1979—1985		0.082*** (0.022)		−0.046** (0.023)
1986—1992		0.105*** (0.022)		−0.028 (0.023)
1993—1999		0.142*** (0.022)		−0.017 (0.024)
2000—2006		0.163*** (0.024)		−0.003 (0.025)
Constant	0.136*** (0.004)	0.029 (0.021)	0.138*** (0.004)	0.162*** (0.023)
Observations	167,475	167,475	151,133	151,133

Models displayed in column (2) and (4) also include the transformed period effects, which are provided in the Appendix, Table A15. Standard errors in parentheses; * *p* < 0.10, ** *p* < 0.05, *** *p* < 0.01.

Notes: The Figures show the values of the cohort dummies plus the constant of our age, period cohort regression model, following Deaton and Paxson [11] with long-term health condition as the dependent variable and as independent variables a full set of age and transformed year dummies. Birth cohort 1922–1929 is the reference. The light lines display 95% confidence intervals. Sources: HILDA data.

**Fig. 2:**
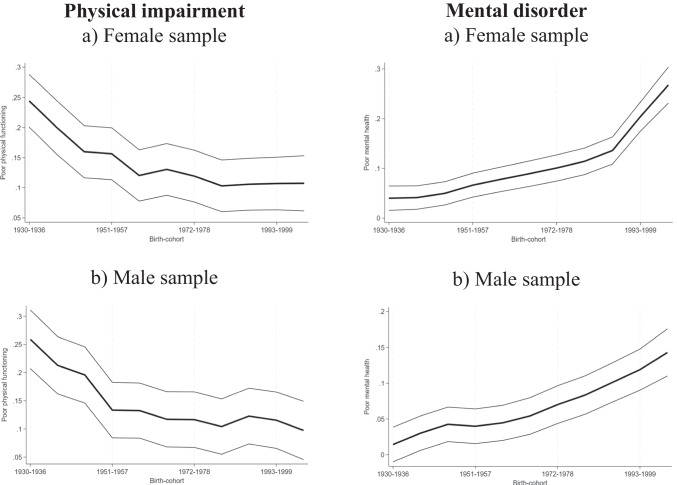
Plotted birth cohort effects of physical impairment and mental disorder prevalence rates for females and males after controlling for age and period. Notes: The Figures show the values of the cohort dummies plus the constant of our age, period cohort regression model, following Deaton and Paxson [11] with physical impairment and probable mental disorder as the dependent variables and as independent variables a full set of age and transformed year dummies. Birth cohort 1922–1929 is the reference. The light lines display 95% confidence intervals. Source: HILDA data

In Table 2 we present results for our two alternative health need proxies, (i) physical impairment in columns (1) and (2), and (ii) probable mental disorder in columns (3) and (4) for females and males respectively. Results show an increase in the prevalence of probable mental disorders among more recently born birth cohorts and at the same time a reduction in physical impairment prevalence rates. Particularly among females, the mental disorder estimates seem to be quite pronounced among the last two birth cohorts of our sample, those born between 1993 to 1999 and 2000 to 2006, with higher magnitudes than the reduction in physical impairment among those cohorts, which may explain the overall increase in long-term health conditions. The opposite is true for males, the reduction in physical impairment prevalence rates is larger than the corresponding increase in mental disorder prevalence rates. The number of observations is slightly reduced in these samples since not all respondents completed the self-completion questionnaire. Cohort effects are plotted in Fig. 2.

**Table 2:** Age and cohort effects on physical impairment and probable mental disorder among male and female respondents in HILDA between 2001 and 2021

	Physical Impairment		Probable Mental Disorder	
	(1)	(2)	(3)	(4)
	Female	Male	Female	Male
Birth year				
Reference group: Respondents born before 1930				
1930–1936	−0.018 (0.022)	−0.013 (0.027)	0.002 (0.013)	0.002 (0.012)
1937–1943	−0.062*** (0.023)	−0.059** (0.026)	0.003 (0.012)	0.017 (0.012)
1944–1950	−0.102*** (0.022)	−0.076*** (0.025)	0.012 (0.012)	0.030** (0.012)
1951–1957	−0.106*** (0.022)	−0.138*** (0.025)	0.028** (0.012)	0.027** (0.012)
1958–1964	−0.142*** (0.022)	−0.139*** (0.025)	0.040*** (0.013)	0.032** (0.013)
1965–1971	−0.132*** (0.022)	−0.155*** (0.025)	0.051*** (0.013)	0.042*** (0.013)
1972–1978	−0.143*** (0.022)	−0.155*** (0.025)	0.063*** (0.013)	0.057*** (0.013)
1979–1985	−0.159*** (0.022)	−0.168*** (0.025)	0.076*** (0.014)	0.071*** (0.014)
1986–1992	−0.156*** (0.022)	−0.149*** (0.025)	0.098*** (0.014)	0.088*** (0.014)
1993–1999	−0.155*** (0.022)	−0.156*** (0.026)	0.166*** (0.015)	0.106*** (0.015)
2000–2006	−0.155*** (0.023)	−0.175*** (0.026)	0.230*** (0.018)	0.130*** (0.017)
Age group				
Reference group: Respondents below 22 years				
22–28	−0.011*** (0.003)	−0.011*** (0.004)	0.012** (0.005)	0.022*** (0.004)
29–35	−0.007 (0.005)	−0.013*** (0.005)	0.029*** (0.006)	0.036*** (0.006)
36–42	0.003 (0.006)	−0.000 (0.006)	0.041*** (0.007)	0.045*** (0.007)
43–49	0.042*** (0.007)	0.015** (0.007)	0.049*** (0.007)	0.053*** (0.007)
50–56	0.086*** (0.008)	0.045*** (0.008)	0.055*** (0.008)	0.055*** (0.008)
57–63	0.142*** (0.009)	0.092*** (0.009)	0.056*** (0.009)	0.052*** (0.008)
64–70	0.202*** (0.011)	0.120*** (0.011)	0.046*** (0.009)	0.044*** (0.009)
71–77	0.312*** (0.013)	0.201*** (0.013)	0.050*** (0.010)	0.053*** (0.010)
78–84	0.476*** (0.016)	0.348*** (0.017)	0.063*** (0.011)	0.068*** (0.011)
85–91	0.615*** (0.022)	0.553*** (0.027)	0.078*** (0.013)	0.095*** (0.014)
92 +	0.663*** (0.041)	0.618*** (0.071)	0.093*** (0.029)	0.130*** (0.050)
Constant	0.262*** (0.022)	0.272*** (0.025)	0.038*** (0.014)	0.013 (0.014)
Observations	151,101	132,770	152,127	133,993

All regressions also include the transformed period effects, which are provided in the Appendix, Table A15. Standard errors in parentheses; * *p* < 0.10, ** *p* < 0.05, *** *p* < 0.01.

### Future prevalence estimates

In Table 3, we compare predicted future prevalence rates of long-term health conditions among females and males using (i) age estimates, and (ii) age and cohort estimates, applied to population projections from the Australian Bureau of Statistics. We find that the projected number of people with long-term health conditions is lower among males and larger among females when using age and birth cohort estimates together, compared to when applying age effects only. While the difference seems small in relative size, with seven percent by 2028 for the female sample and one percent for the male sample, when multiplying this with population numbers, focusing on age-only estimates, would lead to an underestimation of 269,525 females and an overestimation of 36,478 males experiencing a long-term health condition by the year 2028. This increases for females and reduces for males by 2035.

**Table 3:** Predicted prevalence rates of persons with long-term health conditions for 2021,2028 and 2035

	Females			Males		
	2021	2028	2035	2021	2028	2035
Population projections	9,765,534	10,857,303	11,926,971	9,356,182	10,381,046	11,383,295
*Estimates of persons with long-term health condition*						
Age effects only	3,250,867	3,704,158	4,161,159	2,943,699	3,345,143	3,728,761
As % of total population	0.333	0.341	0.349	0.315	0.322	0.328
Age and cohort effects	3,357,066	3,973,683	4,605,415	2,906,774	3,308,666	3,700,577
As % of total population	0.344	0.366	0.386	0.311	0.319	0.325
Difference in pop numbers	106,199	269,525	444,256	−36,925	−36,478	−28,184
Difference as percentage	0.032	0.073	0.107	−0.013	−0.011	−0.001

Source: Australian Bureau of Statistics, Population Projections, Australia, 2017–2066 and author’s calculation based on the HILDA data

Tables 4 and 5 show results for the predicted prevalence rates of persons with physical impairment and probable mental disorder respectively, comparing (i) age only with (ii) age and cohort estimates. Incorporating not only age but also cohort effects into future prevalence estimates results in a decrease in physical impairment estimates and an increase in mental disorder prevalence estimates. Prevalence rates of physical impairment are predicted to be lower if including cohort effects compared to focusing on age effects only, with a reduction of 10.3 percent points for females and 14.4 percent for males by 2035. At the same time, particularly for females, mental disorder prevalence rates are predicted to be larger when adjusting for cohort effects. Predictions for 2035 are 45.2 percent larger compared to when only using age effects, resulting in 741,899 more females who are expected to suffer from mental disorders by 2035. It is 27.4 percent, i.e. 351,721 males by 2035.

**Table 4:** Predicted prevalence rates of persons with physical impairment for 2021,2028 and 2035

	Females			Males		
	2021	2028	2035	2021	2028	2035
Population projections	9,765,534	10,857,303	11,926,971	9,356,182	10,381,046	11,383,295
Estimates of persons with physical impairment						
Age effects only	2,500,881	2,872,570	3,250,475	1,990,214	2,279,269	2,562,861
As % of total population	0.256	0.265	0.273	0.213	0.220	0.225
Age and cohort effects	2,367,754	2,633,692	2,915,162	1,855,028	2,024,737	2,194,066
As % of total population	0.242	0.243	0.244	0.198	0.195	0.193
Difference in pop numbers	−133,127	−238,879	−335,313	−135,186	−254,532	−368,795
Difference as percentage	−0.053	−0.083	−0.103	−0.068	−0.112	−0.144

Source: Australian Bureau of Statistics, Population Projections, Australia, 2017–2066 and author’s calculation based on the HILDA data

**Table 5:** Predicted prevalence rates of persons with probable mental disorder for 2021,2028 and 2035

	Females			Males		
	2021	2028	2035	2021	2028	2035
Population projections	9,765,534	10,857,303	11,926,971	9,356,182	10,381,046	11,383,295
Estimates of persons with probable mental disorder						
Age effects only	1,346,135	1,492,967	1,639,579	1,036,560	1,155,848	1,281,574
As % of total population	0.138	0.138	0.137	0.111	0.111	0.113
Age and cohort effects	1,454,442	1,899,436	2,381,478	1,098,360	1,359,723	1,633,294
As % of total population	0.149	0.175	0.200	0.117	0.131	0.143
Difference in pop numbers	108,307	406,468	741,899	61,800	203,876	351,721
Difference as percentage	0.080	0.272	0.452	0.060	0.176	0.274

Source: Australian Bureau of Statistics, Population Projections, Australia, 2017–2066 and author’s calculation based on the HILDA data

### Estimating future health service requirements: the case of mental disorders

Estimating future mental health service requirements for 2028 and 2035, we discuss the results of applying (i) age only, versus (ii) age- and cohort- prevalence predictions, to (iii) historical use, versus (iv) evidence-based care resource guidelines. Table A13 shows the age- and gender-specific prevalence of respondents who visited a psychiatrist or psychologist at least once in the previous 12 months based on data from the HILDA sample in columns (1) and (2) as well as data from the National Study of Mental Health and Wellbeing (NSMHW) in columns (3) and (4) [3]. Despite varying age groupings between the surveys, the use rates are very similar, providing confidence that we can apply the average number of consultations as historical use estimates.

In Table 6 columns (1)—(4) these historical use estimates are multiplied by the estimated number of people who are predicted to require mental health services for 2028 and 2025. Using age-only predictions, we estimate that in 2028, 1.5 million females and 1.2 million males are expected to visit a mental health professional. Applying the average number of consultations of 5.7 from the NSMHW this results in a total of 8.5 million and 6.6 million consultations, for females and males respectively. Applying guideline best care requirements, this number almost doubles to 14.9 and 11.6 million consultations in 2028.

**Table 6:** Mental health service use predictions by age group, gender and year for constant use versus needs-based models

	Age-constant model				Dynamic needs-based model			
	Female		Male		Female		Male	
	(1)	(2)	(3)	(4)	(5)	(6)	(7)	(8)
Age groups	2028	2035	2028	2035	2028	2035	2028	2035
Estimated number of people who need care								
22–28	198,314	216,947	155,843	170,617	382,951	418,932	233,764	255,926
29–35	222,827	227,558	174,093	178,638	350,802	456,654	234,647	278,054
36–42	215,808	231,449	162,130	178,300	261,630	388,391	209,477	258,772
43–49	185,846	214,358	142,857	162,795	213,330	279,270	174,745	223,843
50–56	162,391	183,285	122,514	137,573	180,950	221,251	140,497	175,437
57–63	149,760	153,848	105,487	110,314	162,054	180,249	111,754	133,251
64–70	123,369	127,554	87,147	88,554	130,751	147,177	87,147	98,504
71–77	100,505	115,577	72,905	83,277	99,646	126,444	72,129	86,820
78–84	78,143	92,485	57,207	65,126	70,080	94,687	60,476	66,987
85–91	40,879	57,736	28,884	42,052	34,747	52,787	27,352	43,963
92 +	15,126	18,786	46,782	64,326	12,495	15,635	7,735	11,736
Total number of people	1,492,967	1,639,579	1,155,848	1,281,574	1,899,436	2,381,478	1,359,723	1,633,294
Historical use estimates								
Total number of consults	8,509,912	9,345,600	6,588,334	7,304,972	10,826,785	13,574,425	7,750,421	9,309,776
Guidelines best care estimates								
Total number of consults	14,929,670	16,395,790	11,558,480	12,815,740	18,994,360	23,814,780	13,597,230	16,332,940
Difference in consults								
Historical use vs guidelines	6,419,758	7,050,190	4,970,146	5,510,768	8,167,575	10,240,355	5,846,809	7,023,164
Age only vs age-cohort					4,064,690	7,418,990	2,038,750	3,517,200
As share					0.27	0.45	0.18	0.27

Using our age- and cohort- estimates of probable mental disorder prevalence rates, multiplied with population projections in columns (5) to (8), we estimate that in 2028, 1.9 million females and 1.4 million males will need to see a mental health professional. Applying historical use estimates that result in future service predictions of 10.8 and 7.8 million consultations, compared to 8.5 and 6.6 million if using age-only models, an increase of around 18 to 27 percent. Using current guidelines that recommend ten consultations with a mental health professional, this would result in service requirement estimates of 14.9 and 11.6 million consults when using age-constant models compared to 19.0 and 13.6 million consults using age-cohort models. An increase of 75 percent. We find that the difference between age only and age- and cohort estimates widens by 2035, leading to an underestimation of 45 percent for females and 27 percent for males, presented in columns (3)—(4) and (7)—(8).

## Discussion and conclusion

In this paper, we outline the flaws of planning methods that rely on current or past health service use by age group and gender and project these estimates onto future population numbers when planning health service requirements in the future. Using Australian survey data, we provide evidence that this method results in a substantive over- or overestimation of health service need requirements, depending on the condition, compared to when using a method that integrates age and birth-cohort changes in health needs and combines these estimates with evidence-based care guidelines.

Our results show that the relationship between health needs and age is not constant across birth cohort, even when accounting for random effects and period effects. We find significant health differences across birth cohorts. While some conditions seem to be declining in age-specific incidence rates among more recently born birth cohorts, others are increasing. Most importantly, when comparing the predictions of our needs-based model to a model based on age-specific use only, we find that estimates differ substantially between methods and that age-constant use methods either over- or under-estimate future health needs, depending on the condition studied. The advantage of our needs-based approach is that it accounts for predicted changes in needs, not only between age groups but also between birth cohorts.

Our results are comparable to findings in Canada and England, where estimates between models that incorporated birth cohort changes in health needs compared to those that assume age constant need, differ substantially [21, 30]. However, while results in these countries indicated a decline of long-standing illnesses among more recently born birth cohorts, we find that the prevalence of long-term health conditions is increasing among females. One reason could be the way survey questions are framed and whether respondents identified mental health conditions as ‘long-standing illnesses’. In the case of HILDA, these are specifically classified as long-term health conditions and seem to be the driving factor in the prevalence increase. This is in line with recent findings that study generational differences in mental health trends in Australia [5]. Using the same dataset, the authors find that mental health is deteriorating among people born in the 1990 s and 1980 s and that mental health is not necessarily worsened by age.

There are a number of limitations to the approach taken. First, we applied evidence-based care guidelines to predict future health service needs. Guidelines often change and advancement in the delivery of care, e.g. through new technology or updated evidence, will need to be incorporated into future planning. In addition, although in a best-case scenario, those in need also take up service offerings, this is unlikely to be the case, and some services may be under-utilised in practice. Second, using responses from the SF-36 questionnaire to identify respondents who are likely to suffer from a mental health disorder has the disadvantage that QoL instruments are subject to reporting heterogeneity, particularly between generations, which may bias our cohort estimates. An analysis that corrects for generational differences in reporting of the latent mental health state would be a further innovation to make in the future. Third, while people with mental disorders also often suffer from co-morbidities, we use a simplified planning model for mental health services and neglect services to other health care professionals. Once planning for other services, such as GP visits or other specialists, controlling for co-morbidities will be important. Fourth, while we try our best to find a health need proxy that is independent of use, we recognize that QoL measures as well as self-reported long-term health conditions may potentially be influenced by previous health care use. In particular for mental health conditions, anti-depressant use may result in relatively high QoL scores, while at the same time, some people might not know they are in need of mental health services. Lastly, our approach relies on the availability of longitudinal, age-specific health data from nationally representative surveys. While such data are not yet available in every country, they are increasingly being collected, and comparable datasets already exist in several settings. In this context, elements of the needs-based planning approach have already been explored in countries such as Canada and the UK [21, 30].

Besides these limitations, our study has several strengths. Compared to previous studies in the field, we go one step further and provide an illustrative application to mental health services, estimating resource requirements in the future, using data from HILDA, and combining it with guidelines best care requirements, and population projections. We find that age-constant use models underestimate mental health service requirements by 2028 and 2035 substantially, whereas the needs-based models show that care needs will most likely be 18 to 45 percent times larger than predictions from traditional health service planning methods. While we provide a proof-of-concept case study using mental disorders to predict future mental health service requirements, we are not able to cover all or even several conditions in a single research paper. Ultimately, planners should repeat this estimation for each condition or illness and then aggregate the findings to plan for future health service and workforce requirements.

In summary, this paper highlights a critical limitation in current health service planning: the reliance on historical, age-specific service use that fails to account for generational shifts in health needs. By incorporating birth cohort effects into our model, we demonstrate that future health service demands, may be significantly over- or underestimated if planners assume stable age-specific patterns of need. Our approach offers a more responsive and forward-looking framework by integrating longitudinal trends and generational differences, allowing for more accurate projections of future service requirements. This model provides a valuable tool for policymakers and planners aiming to align health services with evolving population needs, ensuring that resource allocation keeps pace with demographic and epidemiological change.

## Data Availability

We use data from the Household, Income and Labour Dynamics in Australia (HILDA) survey which we are not allowed to forward or publish. However, upon individual registration (which is simple and not restricted), the data are available to each interested researcher worldwide.

## Appendices

### A. Variable definitions

**Table A1:** Variable definitions for health need proxies

Health need proxy	Definition	Mean	SD
Long-term health condition	= 1 if respondents report to have a long-term health condition, impairment or disability (such as one of the below) that restrict them in their everyday activities, and has lasted or is likely to last, for 6 months or more; = 0 otherwise.	0.28	0.45
	• Sight problems not corrected by glasses / lenses • Hearing problems • Speech problems • Blackouts, fits or loss of consciousness • Difficulty learning or understanding things • Limited use of arms or fingers • Difficulty gripping things • Limited use of feet or legs • A nervous or emotional condition which requires treatment • Any condition that restricts physical activity or physical work (e.g. back problems, migraines) • Any disfigurement or deformity • Any mental illness which requires help or supervision • Shortness of breath or difficulty breathing • Chronic or recurring pain • Long-term effects as a result of a head injury, stroke or other brain damage • A long-term condition or ailment which is still restrictive even though it is being treated or medication is being taken for it • Any other long-term condition such as arthritis, asthma, heart disease, dementia, etc		
Physical functioning	The SF-36 physical functioning score (0–100)	83.13	23.61
Mental health	The SF-36 mental health score (0–100)	73.42	17.54
Physical impairment	= 1 if physical functioning score < 68; = 0 otherwise.	0.19	0.39
Probable mental disorder	= 1 if mental health score < 52; = 0 otherwise.	0.11	0.31

### B. Population projections and sample sizes

**Table A2:** Female population numbers and projections 2000 to 2035

Age group	2000	2007	2014	2021	2028	2035
22–28	946,579	1,010,564	1,199,382	1,322,922	1,367,682	1,496,186
29–35	1,014,458	1,031,309	1,185,052	1,391,882	1,505,588	1,537,556
36–42	1,051,307	1,066,567	1,120,986	1,272,827	1,478,134	1,585,270
43–49	950,846	1,074,723	1,122,084	1,156,824	1,308,772	1,509,565
50–56	824,065	948,347	1,088,228	1,128,235	1,159,937	1,309,179
57–63	585,257	808,029	940,288	1,080,952	1,117,613	1,148,084
64–70	484,788	565,584	780,066	917,937	1,054,440	1,090,203
71–77	441,991	441,817	524,523	727,426	859,015	987,840
78–84	297,075	354,838	369,468	443,675	620,181	734,005
85–91	140,551	177,948	225,331	237,130	291,993	412,402
92+	33,533	48,265	64,961	85,724	93,948	116,681
Total	6,770,450	7,527,991	8,620,369	9,765,534	10,857,303	11,926,971

Source: Australian Bureau of Statistics, Population Projections, Australia, 2017–2066

**Table A3:** Male population numbers and projections 2000 to 2035

Age group	2000	2007	2014	2021	2028	2035
22–28	953,861	1,042,341	1,226,950	1,366,612	1,416,750	1,551,067
29–35	999,608	1,024,501	1,188,185	1,364,605	1,513,854	1,553,375
36–42	1,039,821	1,052,674	1,111,074	1,252,103	1,434,775	1,577,878
43–49	940,899	1,055,572	1,089,700	1,134,143	1,275,508	1,453,523
50–56	842,228	936,841	1,060,155	1,074,503	1,123,979	1,262,140
57–63	599,851	810,143	910,522	1,029,335	1,044,428	1,092,222
64–70	467,140	555,445	767,216	860,992	979,177	994,993
71–77	374,506	397,728	492,065	686,597	775,582	885,923
78–84	196,077	263,122	297,729	380,864	544,832	620,251
85–91	66,094	94,791	137,918	164,415	218,818	318,575
92+	10,944	16,132	25,924	42,013	53,343	73,348
Total	6,491,029	7,249,290	8,307,438	9,356,182	10,381,046	11,383,295

Source: Australian Bureau of Statistics, Population Projections, Australia, 2017–2066

**Table A4:** Sample sizes for females in the HILDA survey periods 2001 – 2021 across age groups and birth cohorts

Birth cohort	Age group												
	15–21	22–28	29–35	36–42	43–49	50–56	57–63	64–70	71–77	78–84	85–91	92+	Total
1923–1929									1,302	2,005	1,218	235	4,760
1930–1936								1,520	2,649	2,193	690		7,052
1937–1943							1,849	3,351	3,461	1,379			10,040
1944–1950						2,555	4,690	5,420	2,410				15,075
1951–1957					3,189	5,751	6,305	2,796					18,041
1958–1964				3,681	6,740	7,305	3,343						21,069
1965–1971			3,244	6,383	7,098	3,222							19,947
1972–1978		2,595	5,729	6,807	3,298								18,429
1979–1985	2,790	5,819	7,370	3,352									19,331
1986–1992	6,788	8,639	4,389										19,816
1993–1999	7,213	3,872											11,085
2000–2006	2,830												2,830
Total	19,621	20,925	20,732	20,223	20,325	18,833	16,187	13,087	9,822	5,577	1,908	235	167,475

Source: Authors’ calculation based on the HILDA data

**Table A5:** Sample sizes for males in the HILDA survey periods 2001 – 2021 across age groups and birth cohorts

Birth cohort	Age group												
	15–21	22–28	29–35	36–42	43–49	50–56	57–63	64–70	71–77	78–84	85–91	92+	Total
1923–1929									1,180	1,433	757	79	3,449
1930–1936								1,361	2,273	1,900	531		6,065
1937–1943							1,837	3,125	3,024	1,105			9,091
1944–1950						2,363	4,248	4,634	1,942				13,187
1951–1957					2,847	4,866	5,335	2,413					15,461
1958–1964				3,260	6,285	6,821	3,110						19,476
1965–1971			2,885	5,593	6,240	2,804							17,522
1972–1978		2,306	4,957	6,045	2,940								16,248
1979–1985	2,718	5,563	6,982	3,151									18,414
1986–1992	6,578	8,151	3,919										18,648
1993–1999	7,092	3,533											10,625
2000–2006	2,947												2,947
Total	19,335	19,553	18,743	18,049	18,312	16,854	14,530	11,533	8,419	4,438	1,288	79	151,133

Source: Authors’ calculation based on the HILDA data

**Table A6:** An overview of age group, cohort and year combinations

Age group	2000	2007	2014	2021	2028	2035
22–28	1972–1978	1979–1985	1986–1992	1993–1999	2000–2006	2007–2014
29–35	1965–1971	1972–1978	1979–1985	1986–1992	1993–1999	2000–2006
36–42	1958–1964	1965–1971	1972–1978	1979–1985	1986–1992	1993–1999
43–49	1951–1957	1958–1964	1965–1971	1972–1978	1979–1985	1986–1992
50–56	1944–1950	1951–1957	1958–1964	1965–1971	1972–1978	1979–1985
57–63	1937–1943	1944–1950	1951–1957	1958–1964	1965–1971	1972–1978
64–70	1930–1936	1937–1943	1944–1950	1951–1957	1958–1964	1965–1971
71–77		1930–1936	1937–1943	1944–1950	1951–1957	1958–1964
78–84			1930–1936	1937–1943	1944–1950	1951–1957
85–91				1930–1936	1937–1943	1944–1950
92+					1930–1936	1937–1943

### C. Descriptive statistics

**Table A7:** Percentage of female HILDA respondents with long–term health conditions between 2001–2021 across age groups and birth cohorts

Birth cohort	Age group												
	15–21	22–28	29–35	36–42	43–49	50–56	57–63	64–70	71–77	78–84	85–91	92+	Total
1923–1929									0.50	0.66	0.80	0.84	0.66
1930–1936								0.42	0.57	0.68	0.72		0.59
1937–1943							0.38	0.46	0.58	0.65			0.51
1944–1950						0.29	0.40	0.46	0.53				0.42
1951–1957					0.23	0.33	0.43	0.49					0.37
1958–1964				0.16	0.23	0.31	0.36						0.27
1965–1971			0.14	0.18	0.25	0.32							0.22
1972–1978		0.13	0.17	0.20	0.25								0.19
1979–1985	0.12	0.13	0.16	0.18									0.15
1986–1992	0.14	0.17	0.18										0.16
1993–1999	0.15	0.18											0.16
2000–2006	0.18												0.18
Total	0.15	0.15	0.16	0.18	0.24	0.32	0.40	0.46	0.55	0.67	0.76	0.82	0.29

Source: Authors’ calculation based on the HILDA data

**Table A8:** Percentage of male HILDA respondents with long–term health conditions between 2001–2021 across age groups and birth cohorts

Birth cohort	Age group												
	15–21	22–28	29–35	36–42	43–49	50–56	57–63	64–70	71––77	78–84	85–91	92+	Total
1923–1929									0.52	0.67	0.76	0.73	0.64
1930–1936								0.50	0.56	0.65	0.68		0.59
1937–1943							0.45	0.48	0.57	0.63			0.52
1944–1950						0.34	0.42	0.48	0.52				0.44
1951–1957					0.23	0.29	0.38	0.45					0.33
1958–1964				0.21	0.23	0.29	0.34						0.26
1965–1971			0.16	0.18	0.24	0.29							0.21
1972–1978		0.15	0.15	0.19	0.25								0.18
1979–1985	0.12	0.11	0.14	0.16									0.13
1986–1992	0.11	0.15	0.18										0.14
1993–1999	0.14	0.15											0.14
2000–2006	0.15												0.15
Total	0.13	0.14	0.15	0.18	0.24	0.30	0.39	0.48	0.55	0.65	0.74	0.76	0.27

Source: Authors’ calculation based on the HILDA data

**Table A9:** Percentage of female HILDA respondents with physical impairment between 2001 – 2021 across age groups and birth cohorts

Birth cohort	Age group												
	15–21	22–28	29–35	36–42	43–49	50–56	57–63	64–70	71–77	78–84	85–91	92+	Total
1923–1929									0.57	0.71	0.83	0.81	0.70
1930–1936								0.42	0.53	0.67	0.79		0.57
1937–1943							0.33	0.37	0.48	0.61			0.43
1944–1950						0.22	0.29	0.35	0.44				0.33
1951–1957					0.18	0.23	0.30	0.34					0.26
1958–1964				0.10	0.14	0.19	0.24						0.17
1965–1971			0.09	0.10	0.16	0.21							0.14
1972–1978		0.09	0.09	0.11	0.15								0.11
1979–1985	0.08	0.07	0.10	0.09									0.09
1986–1992	0.09	0.08	0.08										0.08
1993–1999	0.10	0.08											0.09
2000–2006	0.10												0.09
Total	0.09	0.08	0.09	0.11	0.16	0.21	0.29	0.36	0.50	0.68	0.81	0.83	0.21

Source: Authors’ calculation based on the HILDA data

**Table A10:** Percentage of male HILDA respondents with physical impairment between 2001 – 2021 across age groups and birth cohorts

Birth cohort	Age group												
	15–21	22–28	29–35	36–42	43–49	50–56	57–63	64–70	71–77	78–84	85–91	92+	Total
1923–1929									0.47	0.53	0.67	0.58	0.54
1930–1936								0.35	0.45	0.54	0.67		0.47
1937–1943							0.30	0.29	0.37	0.48			0.34
1944–1950						0.23	0.26	0.29	0.33				0.28
1951–1957					0.14	0.14	0.21	0.24					0.18
1958–1964				0.10	0.12	0.16	0.19						0.14
1965–1971			0.09	0.09	0.11	0.16							0.11
1972–1978		0.07	0.07	0.11	0.11								0.09
1979–1985	0.07	0.08	0.08	0.08									0.08
1986–1992	0.10	0.10	0.09										0.10
1993–1999	0.11	0.08											0.10
2000–2006	0.09												0.09
Total	0.10	0.09	0.08	0.10	0.12	0.16	0.23	0.29	0.40	0.53	0.69	0.70	0.17

Source: Authors’ calculation based on the HILDA data

**Table A11:** Percentage of female HILDA respondents with probable mental disorder between 2001–2021 across age groups and birth cohorts

Birth cohort	Age group												
	15–21	22–28	29–35	36–42	43–49	50–56	57–63	64–70	71–77	78–84	85–91	92+	Total
1923–1929									0.09	0.08	0.09	0.11	0.08
1930–1936								0.09	0.07	0.07	0.07		0.08
1937–1943							0.08	0.08	0.07	0.09			0.08
1944–1950						0.10	0.10	0.08	0.09				0.09
1951–1957					0.11	0.11	0.11	0.09					0.11
1958–1964				0.12	0.11	0.12	0.13						0.12
1965–1971			0.10	0.12	0.13	0.15							0.12
1972–1978		0.11	0.11	0.12	0.14								0.12
1979–1985	0.13	0.11	0.12	0.14									0.12
1986–1992	0.13	0.14	0.17										0.14
1993–1999	0.19	0.20											0.19
2000–2006	0.27												0.27
Total	0.17	0.14	0.13	0.12	0.12	0.12	0.11	0.08	0.08	0.08	0.08	0.09	0.12

Source: Authors’ calculation based on the HILDA data

**Table A12:** Percentage of male HILDA respondents with probable mental disorder between 2001–2021 across age groups and birth cohorts

Birth cohort	Age group												
	15–21	22–28	29–35	36–42	43–49	50–56	57–63	64–70	71–77	78–84	85–91	92+	Total
1923–1929									0.07	0.08	0.10	0.13	0.08
1930–1936								0.06	0.06	0.06	0.07		0.06
1937–1943							0.09	0.06	0.07	0.08			0.07
1944–1950						0.10	0.09	0.07	0.08				0.08
1951–1957					0.10	0.08	0.07	0.06					0.08
1958–1964				0.10	0.08	0.09	0.09						0.09
1965–1971			0.08	0.08	0.10	0.11							0.09
1972–1978		0.11	0.09	0.11	0.11								0.10
1979–1985	0.09	0.10	0.11	0.13									0.10
1986–1992	0.09	0.11	0.14										0.11
1993–1999	0.11	0.13											0.12
2000–2006	0.14												0.14
Total	0.10	0.11	0.11	0.10	0.10	0.09	0.08	0.07	0.07	0.07	0.08	0.11	0.09

Source: Author’s calculation based on the HILDA data

**Table A13:** Age–specific use in mental health services in HILDA vs National Study on Mental Health and Wellbeing (NSMHW)

% of population who visited a psychiatrist/psychologist in the past year				
	HILDA		NSMHW	
	Females (%) (1)	Males (%) (2)	Females (%) (3)	Males (%) (4)
22–28	14.98	8.41		
29–35	14.80	9.01		
16–34			15.1	7.7
36–42	12.54	9.31		
43–49	11.64	7.67		
50–56	9.80	5.99		
57–63	7.40	4.97		
35–64			6.7	3.8
64–70	4.82	3.57		
71–77	2.33	3.13		
78–84	2.72	2.06		
85–91	1.64	1.39		
65–85			2.0	2.3
92+	0	2.50		

### D. Robustness Check

**Table A14:** Robustness checks using the proxy variable approach

	Long-term health condition		Physical impairment		Probable mental disorder	
	Female	Male	Female	Male	Female	Male
	(1)	(2)	(3)	(4)	(5)	(6)
1930–1936	0.027	0.013	–0.019	–0.013	–0.002	–0.002
	(0.021)	(0.022)	(0.022)	(0.027)	(0.013)	(0.012)
1937–1943	0.044∗∗	0.023	–0.064∗∗∗	–0.059∗∗	–0.004	0.012
	(0.021)	(0.022)	(0.023)	(0.026)	(0.012)	(0.012)
1944–1950	0.039∗	0.008	–0.106∗∗∗	–0.077∗∗∗	0.002	0.022∗
	(0.021)	(0.022)	(0.022)	(0.025)	(0.012)	(0.012)
1951–1957	0.058∗∗∗	–0.038∗	–0.111∗∗∗	–0.139∗∗∗	0.015	0.017
	(0.021)	(0.023)	(0.022)	(0.025)	(0.012)	(0.013)
1958–1964	0.031	–0.044∗	–0.148∗∗∗	–0.140∗∗∗	0.024∗	0.019
	(0.021)	(0.023)	(0.022)	(0.025)	(0.013)	(0.013)
1965–1971	0.042∗	–0.050∗∗	–0.139∗∗∗	–0.156∗∗∗	0.032∗∗	0.026∗
	(0.021)	(0.023)	(0.022)	(0.025)	(0.013)	(0.013)
1972–1978	0.054∗∗	–0.047∗∗	–0.152∗∗∗	–0.157∗∗∗	0.040∗∗∗	0.039∗∗∗
	(0.022)	(0.023)	(0.022)	(0.025)	(0.014)	(0.014)
1979–1985	0.044∗∗	–0.073∗∗∗	–0.170∗∗∗	–0.169∗∗∗	0.051∗∗∗	0.051∗∗∗
	(0.022)	(0.023)	(0.022)	(0.025)	(0.014)	(0.014)
1986–1992	0.065∗∗∗	–0.059∗∗	–0.168∗∗∗	–0.151∗∗∗	0.069∗∗∗	0.065∗∗∗
	(0.022)	(0.023)	(0.022)	(0.025)	(0.014)	(0.014)
1993–1999	0.094∗∗∗	–0.052∗∗	–0.168∗∗∗	–0.158∗∗∗	0.136∗∗∗	0.082∗∗∗
	(0.023)	(0.024)	(0.023)	(0.026)	(0.015)	(0.015)
2000–2006	0.104∗∗∗	–0.044∗	–0.175∗∗∗	–0.178∗∗∗	0.205∗∗∗	0.110∗∗∗
	(0.024)	(0.026)	(0.024)	(0.027)	(0.019)	(0.017)
22–28	0.022∗∗∗	0.012∗∗∗	–0.014∗∗∗	–0.011∗∗∗	0.010∗∗	0.020∗∗∗
	(0.005)	(0.004)	(0.003)	(0.004)	(0.005)	(0.004)
29–35	0.047∗∗∗	0.032∗∗∗	–0.011∗∗	–0.013∗∗∗	0.026∗∗∗	0.033∗∗∗
	(0.006)	(0.006)	(0.005)	(0.005)	(0.006)	(0.006)
36–42	0.071∗∗∗	0.053∗∗∗	–0.003	–0.001	0.035∗∗∗	0.040∗∗∗
	(0.007)	(0.007)	(0.006)	(0.006)	(0.007)	(0.007)
43–49	0.126∗∗∗	0.096∗∗∗	0.036∗∗∗	0.014∗∗	0.039∗∗∗	0.045∗∗∗
	(0.009)	(0.009)	(0.007)	(0.007)	(0.007)	(0.007)
50–56	0.199∗∗∗	0.146∗∗∗	0.078∗∗∗	0.044∗∗∗	0.042∗∗∗	0.045∗∗∗
	(0.010)	(0.010)	(0.008)	(0.008)	(0.008)	(0.008)
57–63	0.278∗∗∗	0.217∗∗∗	0.133∗∗∗	0.091∗∗∗	0.040∗∗∗	0.039∗∗∗
	(0.011)	(0.012)	(0.010)	(0.010)	(0.009)	(0.009)
64–70	0.347∗∗∗	0.287∗∗∗	0.191∗∗∗	0.119∗∗∗	0.026∗∗∗	0.028∗∗∗
	(0.013)	(0.013)	(0.011)	(0.011)	(0.010)	(0.009)
71–77	0.460∗∗∗	0.363∗∗∗	0.300∗∗∗	0.200∗∗∗	0.029∗∗∗	0.036∗∗∗
	(0.014)	(0.015)	(0.013)	(0.014)	(0.010)	(0.010)
78–84	0.596∗∗∗	0.502∗∗∗	0.463∗∗∗	0.346∗∗∗	0.038∗∗∗	0.048∗∗∗
	(0.017)	(0.018)	(0.016)	(0.018)	(0.011)	(0.011)
85–91	0.740∗∗∗	0.623∗∗∗	0.601∗∗∗	0.553∗∗∗	0.050∗∗∗	0.074∗∗∗
	(0.022)	(0.025)	(0.022)	(0.027)	(0.013)	(0.015)
92–older	0.844∗∗∗	0.706∗∗∗	0.643∗∗∗	0.614∗∗∗	0.068∗∗	0.112∗∗
	(0.040)	(0.065)	(0.041)	(0.071)	(0.029)	(0.050)
GDP growth rate	–0.008∗∗∗	–0.007∗∗∗	–0.002∗	–0.000	–0.006∗∗∗	–0.005∗∗∗
	(0.001)	(0.001)	(0.001)	(0.001)	(0.001)	(0.001)
Constant	0.096∗∗∗	0.213∗∗∗	0.280∗∗∗	0.274∗∗∗	0.083∗∗∗	0.049∗∗∗
	(0.022)	(0.024)	(0.023)	(0.026)	(0.015)	(0.015)
Number of observations	167,475	151,133	151,101	132,770	152,127	133,993

Standard errors in parentheses ∗ *p* < 0.10, ∗∗ *p* < 0.05, ∗∗∗ *p* < 0.01

**Table A15:** Transformed year dummies for main age, period, cohort analysis

		Long - term health conditions		Physical impairment		Probable mental disorder	
		(1)	(2)	(3)	(4)	(5)	(6)
		Female	Male	Female	Male	Female	Male
2003 transformed		0.021∗∗∗	0.020∗∗∗	–0.001	0.003	0.003	0.006∗∗
		(0.004)	(0.004)	(0.003)	(0.003)	(0.003)	(0.003)
2004 transformed		–0.004	0.006	–0.003	–0.005	0.007∗∗	0.008∗∗
		(0.003)	(0.004)	(0.003)	(0.003)	(0.003)	(0.003)
2005 transformed		0.019∗∗∗	0.018∗∗∗	–0.000	0.004	0.002	0.002
		(0.004)	(0.004)	(0.003)	(0.003)	(0.003)	(0.003)
2006 transformed		0.004	0.002	–0.002	–0.003	0.003	–0.003
		(0.003)	(0.004)	(0.003)	(0.003)	(0.003)	(0.003)
2007 transformed		0.006	0.005	0.000	–0.002	–0.004	–0.003
		(0.004)	(0.004)	(0.003)	(0.003)	(0.003)	(0.003)
2008 transformed		–0.006	–0.014∗∗∗	–0.010∗∗∗	–0.008∗∗	–0.003	–0.002
		(0.004)	(0.004)	(0.003)	(0.003)	(0.003)	(0.003)
2009 transformed		0.025∗∗∗	0.010∗∗∗	–0.009∗∗∗	–0.006∗	–0.006∗	–0.012∗∗∗
		(0.004)	(0.004)	(0.003)	(0.003)	(0.003)	(0.003)
2010 transformed		0.003	–0.003	0.006∗	0.000	–0.004	–0.009∗∗∗
		(0.004)	(0.004)	(0.003)	(0.003)	(0.003)	(0.003)
2011 transformed		0.002	–0.004	0.004	–0.001	–0.009∗∗∗	–0.006∗∗
		(0.003)	(0.003)	(0.003)	(0.003)	(0.003)	(0.003)
2012 transformed		–0.002	–0.012∗∗∗	0.010∗∗∗	–0.005∗	–0.012∗∗∗	–0.008∗∗∗
		(0.003)	(0.003)	(0.003)	(0.003)	(0.003)	(0.003)
2013 transformed		0.024∗∗∗	0.015∗∗∗	0.011∗∗∗	0.004	–0.010∗∗∗	–0.007∗∗∗
		(0.003)	(0.003)	(0.003)	(0.003)	(0.003)	(0.003)
2014 transformed		–0.000	0.006∗	0.009∗∗∗	0.006∗	–0.007∗∗	–0.004
		(0.003)	(0.003)	(0.003)	(0.003)	(0.003)	(0.003)
2015 transformed		0.001	–0.000	0.001	–0.001	–0.004	–0.003
		(0.003)	(0.003)	(0.003)	(0.003)	(0.003)	(0.003)
2016 transformed		–0.013∗∗∗	–0.013∗∗∗	–0.002	–0.001	–0.004	–0.002
		(0.003)	(0.003)	(0.003)	(0.003)	(0.003)	(0.003)
2017 transformed		0.009∗∗∗	0.010∗∗∗	0.004	0.009∗∗∗	–0.004	–0.005∗∗
		(0.003)	(0.003)	(0.003)	(0.003)	(0.003)	(0.003)
2018 transformed		–0.011∗∗∗	0.002	0.001	0.013∗∗∗	–0.007∗∗	0.001
		(0.003)	(0.003)	(0.003)	(0.003)	(0.003)	(0.003)
2019 transformed		–0.013∗∗∗	–0.004	–0.007∗∗	0.002	0.002	0.000
		(0.003)	(0.003)	(0.003)	(0.003)	(0.003)	(0.003)
2020 transformed		–0.006∗∗	–0.005	–0.008∗∗∗	–0.013∗∗∗	0.017∗∗∗	0.014∗∗∗
		(0.003)	(0.003)	(0.003)	(0.003)	(0.003)	(0.003)
2021 transformed		–0.000	–0.001	–0.005∗	–0.004	0.024∗∗∗	0.017∗∗∗
		(0.003)	(0.003)	(0.003)	(0.003)	(0.003)	(0.003)
Constant		0.029	0.162∗∗∗	0.262∗∗∗	0.272∗∗∗	0.038∗∗∗	0.013
		(0.021)	(0.023)	(0.022)	(0.025)	(0.014)	(0.014)
Number of observations		167,475	151,133	151,101	132,770	152,127	133,993

Standard errors in parentheses ∗ *p* < 0.10, ∗∗ *p* < 0.05, ∗∗∗ *p* < 0.01

## Footnotes

1 Although people with mental disorders do not only seek care from a mental health professional, such as a psychiatrist or psychologist but may also consult with a GP or allied health professional regarding their mental health condition, due to co-morbidities, it is difficult to disentangle which services from these professionals were mental health-specific services, compared to visits to the doctor for other health conditions. In this exercise, we therefore focus on consultations with mental health professionals only. This is in line with Australian care guidelines for the treatment of mental disorders.

2 In the review, mental disorders are grouped into mood disorders (depression, bipolar disorder), anxiety disorders (anxiety, panic disorder, phobia, obsessive–compulsive disorder, posttraumatic stress disorder), substance use disorder, eating disorders, adjustment disorders, and dissociative disorders. The most effective treatment pathways for mental disorders include between eight to twelve sessions with a mental health professional across all six groups, ranging from a minimum of six to a maximum of 24 sessions. The median and as well as the average number of sessions among all studies was ten consultations. This is also in line with the current government subsidies for mental health treatment plans in Australia, where eligible people can claim up to ten sessions with a mental health professional each calendar year. We thus use ten sessions as the guidelines recommended standard.
